# Low Energy Consumption Photoelectric Memristors with Multi-Level Linear Conductance Modulation in Artificial Visual Systems Application

**DOI:** 10.1007/s40820-025-01816-y

**Published:** 2025-07-01

**Authors:** Zhenyu Zhou, Zixuan Zhang, Pengfei Li, Zhiyuan Guan, Yuchen Li, Xiaoxu Li, Shan Xu, Jianhui Zhao, Xiaobing Yan

**Affiliations:** College of Electron and Information Engineering, School of Life Sciences, Institute of Life Science and Green Development, Key Laboratory of Brain-Like Neuromorphic Devices and, Systems of Hebei Province, Hebei University, Baoding, 071002 People’s Republic of China

**Keywords:** Photoelectric memristors, Optical synapses, Low energy, Linear response, Intelligent drive

## Abstract

Optical synapses have an ability to perceive and remember visual information, making them expected to provide more intelligent and efficient visual solutions for humans. As a new type of artificial visual sensory devices, photoelectric memristors can fully simulate synaptic performance and have great prospects in the development of biological vision. However, due to the urgent problems of nonlinear conductance and high-energy consumption, its further application in high-precision control scenarios and integration is hindered. In this work, we report an optoelectronic memristor with a structure of TiN/CeO_2_/ZnO/ITO/Mica, which can achieve minimal energy consumption (187 pJ) at a single pulse (0.5 V, 5 ms). Under the stimulation of continuous pulses, linearity can be achieved up to 99.6%. In addition, the device has a variety of synaptic functions under the combined action of photoelectric, which can be used for advanced vision. By utilizing its typical long-term memory characteristics, we achieved image recognition and long-term memory in a 3 × 3 synaptic array and further achieved female facial feature extraction behavior with an activation rate of over 92%. Moreover, we also use the linear response characteristic of the device to design and implement the night meeting behavior of autonomous vehicles based on the hardware platform. This work highlights the potential of photoelectric memristors for advancing neuromorphic vision systems, offering a new direction for bionic eyes and visual automation technology.

**Supplementary Information:**

The online version contains supplementary material available at 10.1007/s40820-025-01816-y.

## Highlights

- Developed a novel photo-memristor with single-pulse low energy consumption (187 pJ) and multi-pulse linearity up to 0.996.
- Using photoelectric synaptic characteristics, achieved long-term memory in a 3 × 3 array and over 92% activation of female facial feature recognition in a 64 × 64 model.
- Using the continuous response characteristics of optical synapses, an intelligent driving system with automatic night meeting was designed.

**Supplementary Information:**

The online version contains supplementary material available at 10.1007/s40820-025-01816-y.

## Graphical abstract

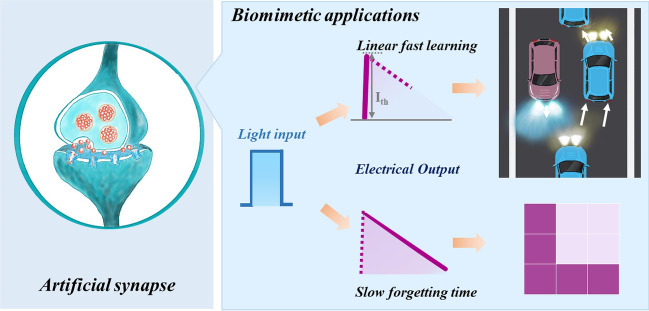

## Introduction

Advanced artificial vision technology plays a crucial role in areas such as smart homes, self-driving cars and humanoid robots [1–4]. These systems are usually designed using complementary metal oxide semiconductor (CMOS)-integrated circuits based on von Neumann architectures, but this design approach faces “memory wall” and “power wall” problems, which limit its development in the field of artificial intelligence. In contrast, the human visual system exhibits a high energy-efficiency ratio, which stems from the retina's ability to instantly perceive and initially process light, coupled with parallel processing mechanisms in the brain's visual cortex, which are able to rapidly and accurately process visual information [5, 6]. For example, synaptic plasticity in the human retina efficiently extracts key visual features [7] and reduces data redundancy, thereby accelerating processing in the visual cortex. In order to mimic this efficient biological vision mechanism, the development of an artificial vision system with sense and memory behavior is the key to realizing the bionic eyes and bionic robots [8].

Recently, artificial vision system has been paid more attention, and there are a variety of implementation methods; there are sensor connected memristor devices, photodetectors and memristors integrated, and three-terminal photoelectric devices [9–12]. The method of series memristor not only increases the complexity of system integration, but also increases the energy consumption, which is not conducive to the large-area integration of vision chips. At present, the three-terminal photoelectric transistors are more studied [10, 11], which has shown the feasibility of neuromorphic vision systems. But due to structural problems, the array density is low and the circuit design is complex, which may lead to low processing speed and high energy consumption [13]. As a new neuromorphic vision device, two-terminal photoelectric memristors can realize the way of processing light information like human eyes and complete the perception, memory and processing of signals [13–16]. More importantly, the two-terminal photoelectric memristors are more conducive to realizing the high density integration of simple cross-point arrays of ultra-high-resolution vision chips [17]. However, although two-terminal photoelectric memristor have great potential in the field of visual information processing, it is difficult for most devices to achieve multistage linear conductance modulation at low energy consumption [18–24], which will limit further applications of devices in high-precision visual perception [25, 26]. Because linear conductance modulation optimizes each pulse increment [26], allowing the photoelectric memristor to be precisely programmed to the target conductance state, thus avoiding redundant weight tuning processes and the need for peripheral circuitry [27], which can reduce system energy consumption. Reducing energy consumption is the continuous pursuit of the whole brain-like research field, dreaming that integrated chips can complete system-level tasks with low energy consumption and high efficiency like human brain [28]. Vision is at the top of the list of ways humans acquire information [28]. The multi-level linear conductance modulation optical synapse based on photoelectric memristor can effectively improve the efficiency of information acquisition and processing, reduce system energy consumption, and promote the further development of vision chips.

The cerium dioxide (CeO_2_) is an excellent photosensitive material and has received extensive attention [29, 30]. Research shows that by introducing defect energy levels during the preparation process, the light response range can be effectively extended from ultraviolet light (UV) to visible light, improving its response capability in optoelectronic devices. The zinc oxide (ZnO) has the advantages of optical transparency, a bandgap width of approximately 3.37 eV, as well as good chemical stability and high resistivity, which helps to reduce power consumption. The CeO_2_/ZnO interface formed by CeO_2_ and ZnO is prone to capturing oxygen vacancies to achieve the accumulation of charges [30]. This accumulation is conducive to the continuous modulation of conductivity and plays a key role in the realization of linear conductivity modulation.

Here, we present photoelectric memristors structured as TiN/CeO_2_/ZnO/ITO/Mica, which dynamically adjusts the barrier height of the CeO_2_/ZnO interface by using light, achieving excellent performance in multi-level linear (99.6%) conductance modulation. And the device has a minimum energy consumption of only 187 pJ at 0.5 V operating voltage, which is extremely low energy consumption and gives it an advantage among visual perception integrated chips. In addition, a variety of typical synaptic plasticity functions of the device based on photomodulation were realized, including paired pulse facilitation (PPF), paired pulse depression (PPD), short-term memory (STM) and long-term memory (LTM), transition from short-term to long-term memory, "learning-experiencing" behavior and "Pavlovian conditioning" behavior. In particular, two aspects of long-term memory and linear multilevel synaptic weight plasticity demonstrate its potential for application in the field of visual perception. Using the above two features, we successfully implemented the recognition and long-term storage of human visual image memory function “L” on a 3 × 3 array and further simulated the image recognition function of specific female facial features with an activation rate of over 92%. In addition, we also designed an intelligent driving system based on linear optical synapse to realize the function of autonomous driving meeting at night. Experiments have demonstrated that the device performs well in simulating complex visual tasks and shows great potential especially for applications in advanced visual perception systems.

## Experimental Section

### Fabrication of Optical Synapses Device

Preparation of ITO bottom electrode on Mica substrate using radio frequency sputtering method. Firstly, pump the back pressure of the magnetron sputtering equipment to 2 × 10^–4^ Pa, then adjust the RF power to 100 W, and adjust the argon oxygen ratio inside the cavity to 5:1. Sputtering is completed under the condition of a sputtering pressure of 0.5 Pa. Preparation of ZnO and CeO_2_ functional layers using radio frequency sputtering method. Firstly, the back pressure of the magnetron sputtering equipment is pumped to 2 × 10^–4^ Pa, and then, the argon oxygen ratio inside the cavity is modulated to 1:2. The preparation of ZnO thin films is completed under the conditions of a sputtering power of 80 W and a sputtering pressure of 3 Pa. Afterward, the sputtering pressure was increased to 3 Pa under pure argon conditions, and the preparation of CeO_2_ thin films was completed under a sputtering power of 80 W. Preparation of TiN top electrode using DC magnetron sputtering method. Firstly, the back pressure of the magnetron sputtering equipment is pumped to 2 × 10^–4^ Pa, and the sputtering pressure is set to 0.8 Pa under pure argon conditions. Using a mask with a diameter of 100 μm, a TiN top electrode with a thickness of about 40 nm is grown by sputtering on a SiO_2_ layer for 60 min.

### Optical and Electrical Measurements

Using a probe station to connect the upper and lower electrodes of the device to form a conductive path and using the Keithley 2400 digital source meter, photoelectric coupling can be effectively achieved, completing various biomimetic characteristics of artificial photoelectric synapses. By combining the DG5072 waveform generator and DS4034 oscilloscope, pulse testing of the device is completed. The function generator is used to write pulses and emit them, and the oscilloscope reflects the test waveform. By setting different pulse parameters, a series of applications such as conductance control of the device can be achieved.

## Results and Discussion

### Role of Optoelectronic Memristor in Optical Synapses

**Fig. 1:**
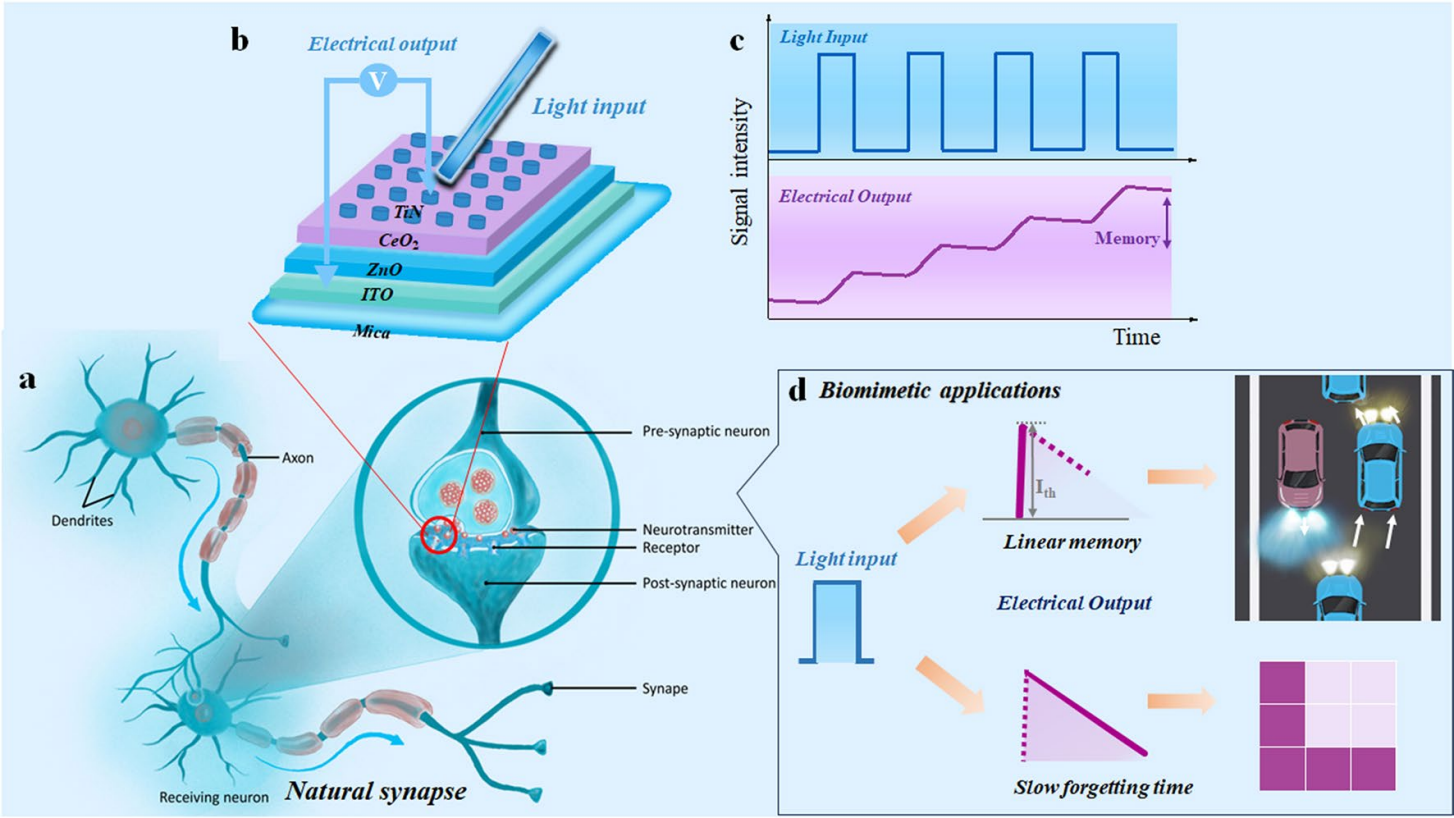
Bioinspired visual system. **a** Schematic diagram of biological synapses. **b** Optical synapses device structured as TiN/CeO_2_/ZnO/ITO/Mica. **c** Optical synapses performance. **d** Biomimetic applications completed based on the devices

In the human visual system, optical information is captured by the retina and converted into neural signals firstly, and these signals are subsequently transmitted to the brain for processing for visual memory and recognition. This process is accomplished primarily through synapses; synapses release neurotransmitters when a nerve impulse arrives, thus accomplishing signaling and processing (Fig. 1a). In order to effectively simulate the visual synapse, we need to introduce light sensing into the memristor. So, we chose CeO_2_ and ZnO materials with excellent optical response [29, 31]; they are both wide-band gap semiconductors that help suppress leakage current and enable the preparation of low-power devices. Therefore, we designed TiN/CeO_2_/ZnO/ITO/Mica structure novel optoelectronic memristor device (Fig. 1b). The device not only exhibits excellent light sensing capability, but also efficiently performs conversion and storage of optoelectronic signals. And it has a good linear photoresponse performance (Fig. 1c), which provides the possibility of visual bionics applications based on conductance modulation with high linearity. For example, it can be applied to specific scenarios that require visual information, such as face recognition and intelligent driving. At the same time, the device's long times forgetting rate enables it to mimic human visual memory for long time storage of images (Fig. 1d), and a further part verifies its application potential in face recognition based on artificial visual characteristics. It provides a new idea for intelligent sensing and machine vision to build an artificial vision system based on photoelectric memristor that integrates sensing, storing and calculating.

### Multi-Level Linear Incremental Conductance Modulation

**Fig. 2:**
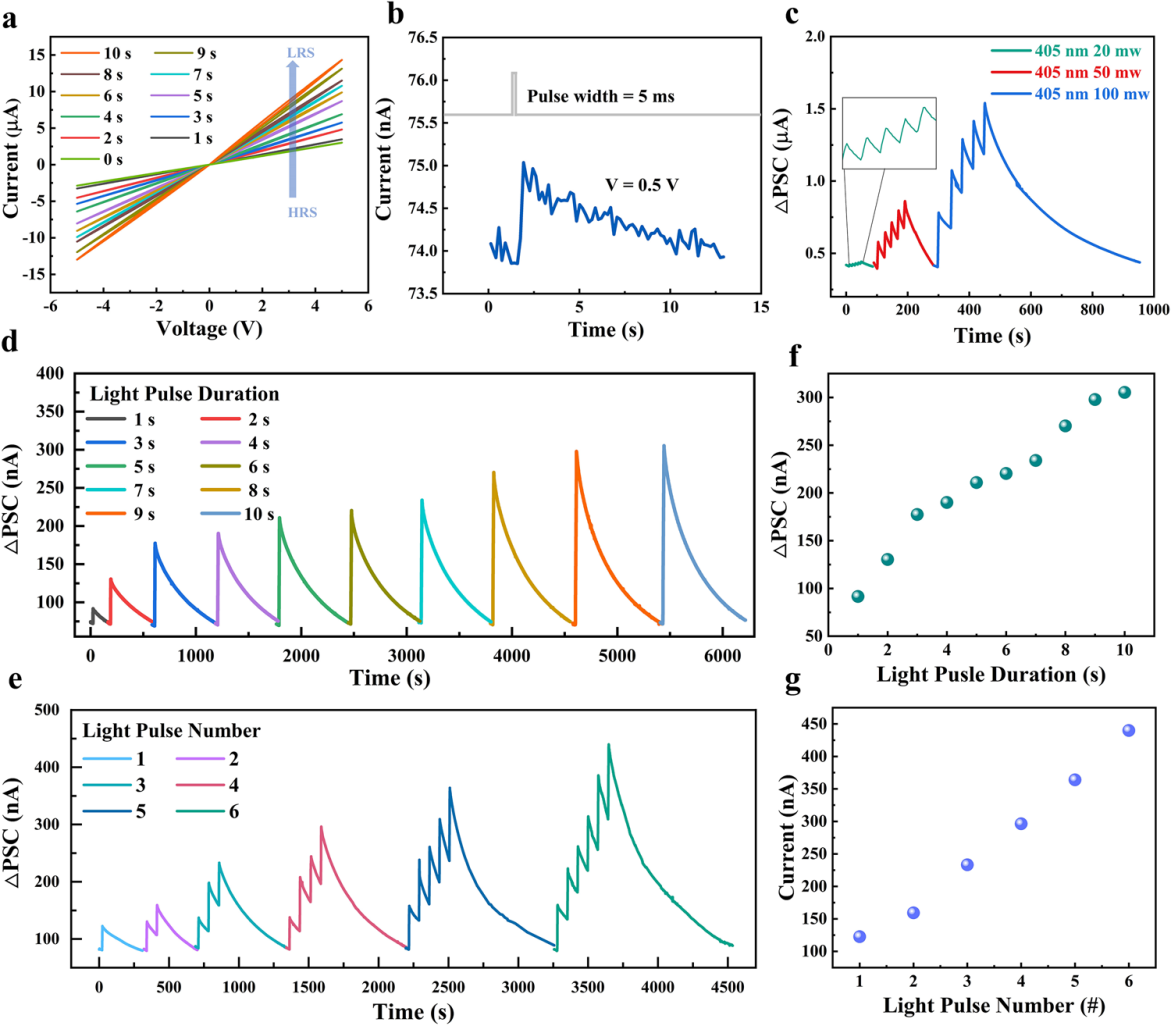
Optical synapses characteristics of TiN/CeO_2_/ZnO/ITO/Mica. **a** *I*–*V* curves at different light times (0–10 s). **b** Power consumption of the device at 0.5 V is based on the equation *E* = *I*_peak_*V*t, where *I*_peak_, *V*, and *t* denote the maximum optical response current, the holding voltage, and the duration of the light pulse, respectively. **c** Response current of the device for different powers (20, 50, and 100 mW) and 5 consecutive optical pulse conditions. **d** Device response current to light pulses of different durations (1–9 s) for the same illumination conditions. **e** PSC response to a series of light pulses with the same duration and different number of pulses (1–6). **f** PSC plotted as a function of light pulse duration. **g** PSC plotted as a function of the number of light pulses

The adaptive response of biological synapses to changes in external stimuli is one of their key features, and the perception of light information is particularly important in the visual domain [17]. Optical synapse can adapt to their environment by adjusting their responses to drastic changes in different light conditions. If the optical synapses are over-sensitive to strong light and easily saturated, or under-responsive to weak light, the accuracy of their recognition will be compromised, a feature that can be captured by the response of the optical synapses to different parameters of light pulses. When the device was illuminated for different times (405 nm, 50 mW), its *I*–*V* curve showed an increasing trend (Fig. 2a). With the increase in light duration, the resistance of the device gradually decreases, which proves that the device has a good continuous light response capability and provides an important foundation for the study of visual perception based on optical synapses. Notably, the realization of low energy consumption is key to the development of ideal artificial optical synapses devices. Conventional complementary metal oxide semiconductor (CMOS) circuits consume approximately 900 pJ per spike [32]; however, the device can achieve a minimum consume approximately of only 187 pJ per spike at 0.5 V operating voltage (Fig. 2b); this energy consumption is significantly lower than conventional CMOS and most current reported neuromorphic devices (Table S1). It is demonstrated that the device has a very high potential for application in the field of constructing low-power artificial optical synapses. The changes in external light stimulation can lead to changes in synapses plasticity, and with increasing light intensity (20, 50, and 100 mW, respectively, as shown in Fig. 2c), light durations (1–10 s, as shown in Fig. 2d), and light pulse number (1–6, pulse width 2 s, interval 30 s, as shown in Fig. 2e), the device's post synaptic current (PSC) gradually increases, and with increasing photocurrent, its decay time also gradually increases, demonstrating good plasticity (Fig. S1). The good linear relationship (the *R*^2^ = 0.969, 0.993 as shown in Fig. 2f, g, respectively.) between PSC and duration and number of light pulses suggests that the photoelectric memristor can achieve good linear control and has better application potential in high-precision scenarios [26], which is necessary for most bio-optical sensors to obtain accurate performance with different functions in information processing [33]. In addition, 8 devices were randomly tested to explore the device variability between different devices, and the response characteristics of each device to light pulses of 1, 5, and 10 were tested separately (Fig. S2), indicating that the optical response of the device is stable. In addition, to the direct light test of the device, the electric pulse test under different light conditions is also carried out (Figs. S3, S4, S5). It can be found that photoelectric hybrid regulation can make the conductance change more accurate; this is because the cerium oxide film has defect levels, which can extend the optical response from ultraviolet light to the visible range [29]. In the state of thermal equilibrium, since the electron affinity of ZnO is higher than that of CeO_2_, electrons in CeO_2_ will transfer to ZnO, resulting in a positive charge at the CeO_2_ interface and a negative charge at the ZnO interface, thereby forming an internal electric field and potential barrier at the interface. When 405 nm visible light is irradiated onto the CeO_2_/ZnO interface, the photon energy is higher than the bandgap width between the two, generating electron–hole pairs at the CeO_2/_ZnO interface. Under the action of the built-in electric field, electrons migrate toward the CeO_2_ side, holes migrate toward the ZnO side, the charge distribution at the interface changes, and the potential barrier height changes accordingly (Fig. S6). Under light excitation, the generated holes are captured by traps at the CeO_2_/ZnO interface, resulting in charge accumulation, a decrease in the potential barrier height, and an increase in the device's conductivity. After the light irradiation stops, the captured holes are gradually recombined, and this process generates the memory characteristics of the light signal in a relatively short period of time. Finally, the potential barrier height is dynamically adjusted through photoelectric hybrid to achieve the linear change of electrical conductivity [29].

**Fig. 3:**
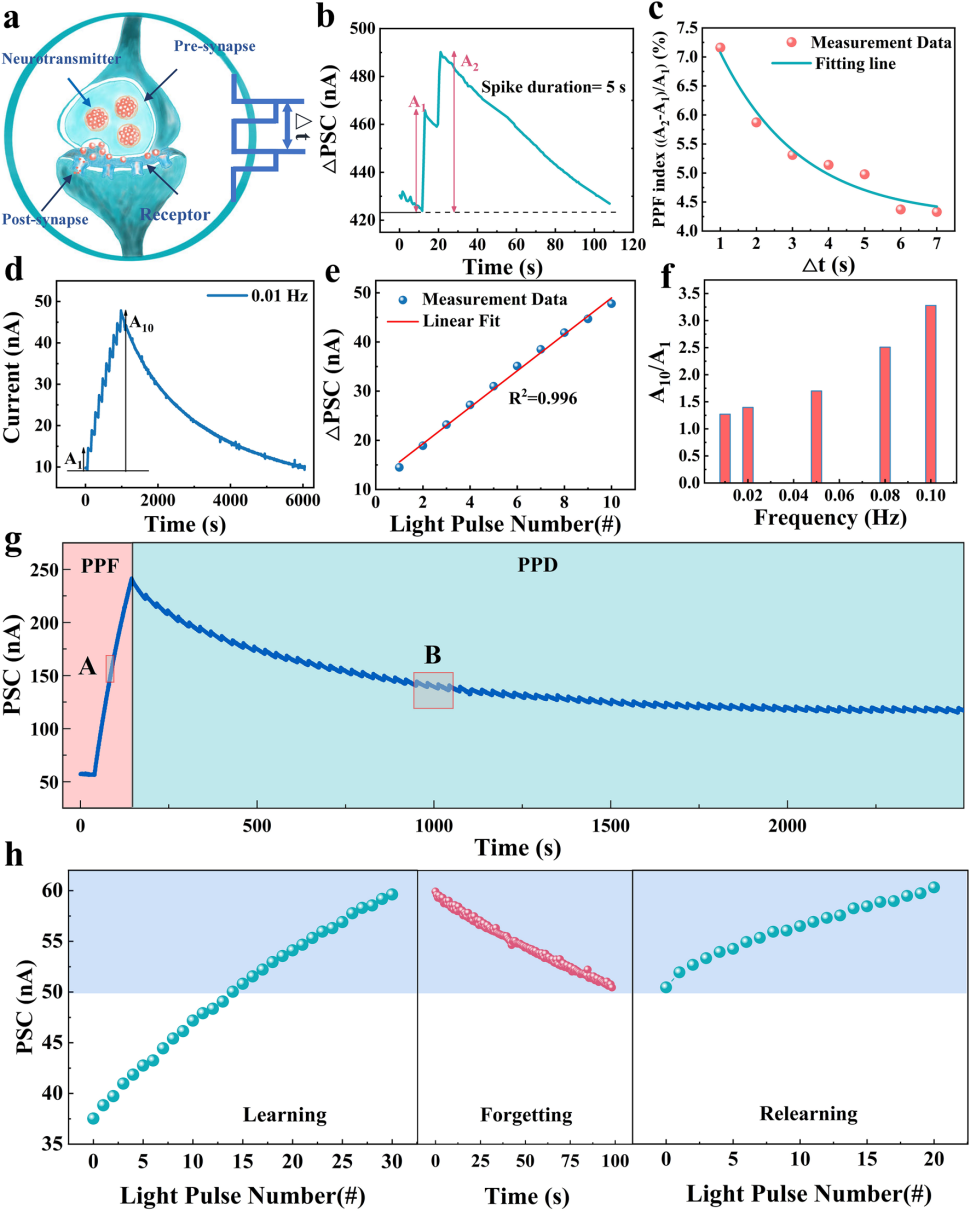
Simulation of PPF and PPD using optical synapses devices. **a** Schematic diagram of a biological synapse triggered by a pair of presynaptic action potentials at time intervals (Δ*t*). **b** Postsynaptic currents triggered by a pair of presynaptic light pulses (50 mW, pulse width 1 s) with a time interval (Δ*t*) of 1 s. *A*_1_ and *A*_2_ denote the amplitudes of the first and second bistable PSCs, respectively. **c** PPF index, defined as *A*_2_/*A*_1,_ was plotted as a function of the optical pulse interval time Δ*t*. The fitted line shows an exponential decrease in the PPF index with increasing *t*. **d** Modulation of the PSC of the device induced by 10 consecutive optical pulses (pulse width 20 s, interval 80 s, frequency 0.01 Hz). *A*_1_ and *A*_10_ denote the magnitude of the change in the PSC at the first and tenth optical pulses. **e** Variation of PSC with respect to the number of light pulses and PSC plotted as a function of the number of light pulses and linear fit (*y* = *ax* + *b*) and *R*^2^ = 0.996, which shows good linearity, *R*^2^ is the coefficient of determination measures the degree of fit between the fitted line and the actual data, and the closer the *R*^2^ value is to 1, the better the linearity [34]. **f** Variation of PSC gain (*A*_10_/*A*_1_) versus optical pulse frequency; **g** PPF to PPD conversion obtained by changing the time interval between pulses; **h** Simulating the “learning-experiencing” behavior of the human brain, fewer stimuli after a learning session can enable the device to recover the memory current after the forgetting process

PPF and PPD are a highly representative behavior in biological synapses, which are mainly characterized by the fact that synapses show enhancement and attenuation of neurotransmitter release when stimulated by continuous nerve impulses; this phenomenon shows its unique application in the encoding of visual time signals [9]. PPF represents the release of synaptic neurotransmitters influenced by the time interval between identical pulses of stimulation (Fig. 3a, b, pulse duration 1 s, interval of 5 s). The PSC triggered by the second optical pulse is significantly higher than the first one, indicating that the device possesses the enhancement capability of the double pulse; this phenomenon may be related to the dynamic behavior of photogenerated carriers [34]. The PPF ratios decay exponentially as ∆*t* increases (Fig. 3c), which indicated the potential of optical synapses in modeling biological learning patterns [35]. The PSC response of the device under stimulation with a sequence of 10 optical pulses at a frequency of 0.01 Hz and a duration of 20 s, which at the tenth pulse (A_10_) is significantly higher than that of any of the previous pulses (Fig. 3d, e), since the photon-induced carriers in the device are not completely consumed at short time intervals, subsequent light pulses cause further linear (*R*^2^ = 0.996) accumulation of carriers. Therefore, its learning and memory abilities can be enhanced. In addition, the PSC gain (A_10_/A_1_) decreases as the pulse frequency decreases, and the PSC response of the device can be converted from PPF to PPD by changing the frequency between optical pulse trains (Figs. 2g and S7, A: the frequency of 0.67 Hz and B: frequency of 0.0328 Hz) [36]. The main reason lies in that when the light exposure time is not changed but only the pulse interval is increased to regulate the pulse frequency, the accumulated charges at the CeO_2_/ZnO interface will recombine under the action of the electric field during the period without light exposure. Secondly, due to the increase in the pulse interval, the consumed charges cannot be replenished in a short time, which confirms that PSCs of photosynapse also have a certain dependence on the frequency of light pulses [36].

In addition, synaptic plasticity is particularly important in the characterization of optical synapses, in which long-term plasticity (LTP), which is formed through the transition between STM and LTM during repetitive learning, underlies memory formation. In fact, repetitive learning activities help the brain process and store information more efficiently, and the strength of these memories is significantly affected by the frequency and intensity of learning. To better model the plasticity of the optical synapses, we achieved device STM and LTM transitions by adjusting the width and number of light pulses (Figs. S8 and S9). Also, synaptic plasticity allows the brain to go through the process of learning, forgetting, and relearning when learning new knowledge. Typically, the time required for re-learning is much shorter than the initial learning, and this behavior is also realized in our device, where after the first learning (sequence of 30 light pulses with a pulse width of 2 s and an interval of 3 s) and forgetting, the device recovers to the previous learning level in 20 light pulses, as shown in Fig. 3h. In addition, the device successfully simulated the behavior of Pavlov's conditioned reflex experiment using this property (Fig. S10), and using this experiment it can be demonstrated again that increasing the learning process of the device improves the memory effect. The above results illustrate the significant advantages of the device in terms of image memory function, which not only broadens the application field of the device, but also provides a strong technical support for the development of future image processing technology.

### Application of Optical Synapses in Visual Perception Scenarios

**Fig. 4:**
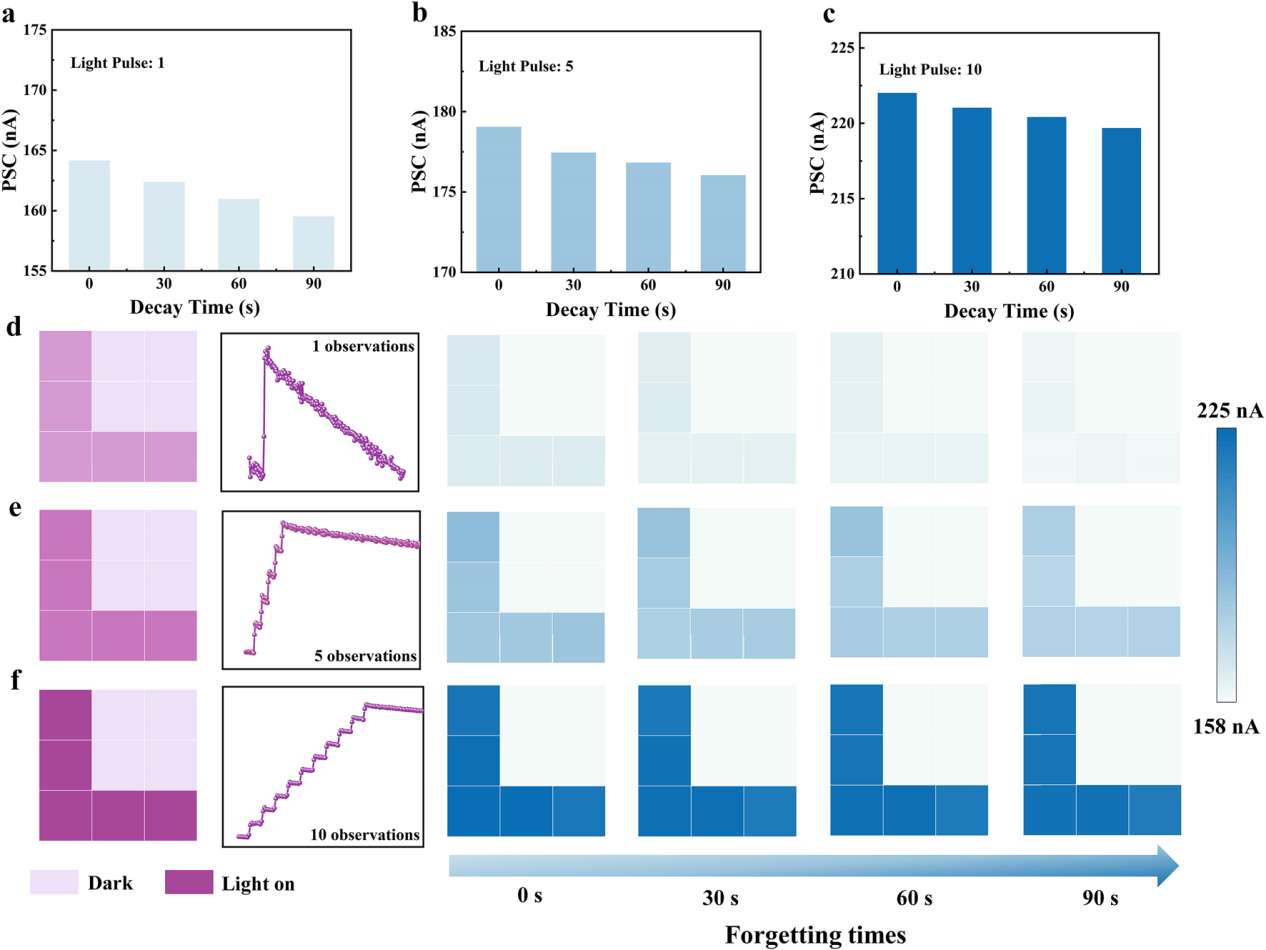
Verification of human visual image memory function. Light response of the device after **a** one observation, **b** two observations, and **c** three observations with different numbers of observations for 90 s forgetting time; **d** Light response image mapping after one observation; **e** three observations and **f** ten observations

In the human visual perceptual system, the more retinal cells observe an image, the greater the synaptic weights in the visual nervous system, which results in the brain remembering the image more clearly and with less likelihood of it being forgotten [24]. We validated the simulation of human visual perception behavior using 3 × 3 arrays of this device (Fig. 4a); the store-and-forget durations for each of the nine devices were collected for different numbers of light pulses. When observing the image once (applying a light pulse to the device once) the PSC of the device is forgotten with time, while gradually increasing the number of observations from 1 to 5 (light pulse width 1 s, interval 5 s) or even 10, the decay of the device's synaptic weights is getting smaller and smaller, which also means that its degree of forgetfulness is getting smaller and smaller (Fig. 4b, c). As the number of observations increases, the synaptic weights between neurons increase and the image is memorized more clearly. To simulate the process of memory and forgetting in the human visual system, an “L” pattern consisting of nine devices was used for visualization, and the changes in the synaptic weights of the devices represented the imaging effects for different numbers of observations. When an observation is made, the “L” character can be clearly displayed, but with the increase of the forgetting time, the image becomes blurred with only a small residue at 90 s (Fig. 4d). The number of image observations is increased to 5 or even 10, the memory level of the image is gradually increased, and the image is still clearly visible when the forgetting time reaches 90 s (Fig. 4e, f). In addition to this, the process of forgetting the image mapping after 10 observations was also explored, which can be found that with the growth of time, the memory level of the image gradually decreases, and the image basically disappears when the forgetting time is as long as 8000 s (Fig. S11). This indicates that the device can acutely perceive and memorize images from optical stimuli, thus realizing a realistic simulation of the human visual perceptual system, and it is able to process visual information more accurately than conventional CMOS image sensors while consuming less power like the processing in the visual cortex of the human brain.

**Fig. 5:**
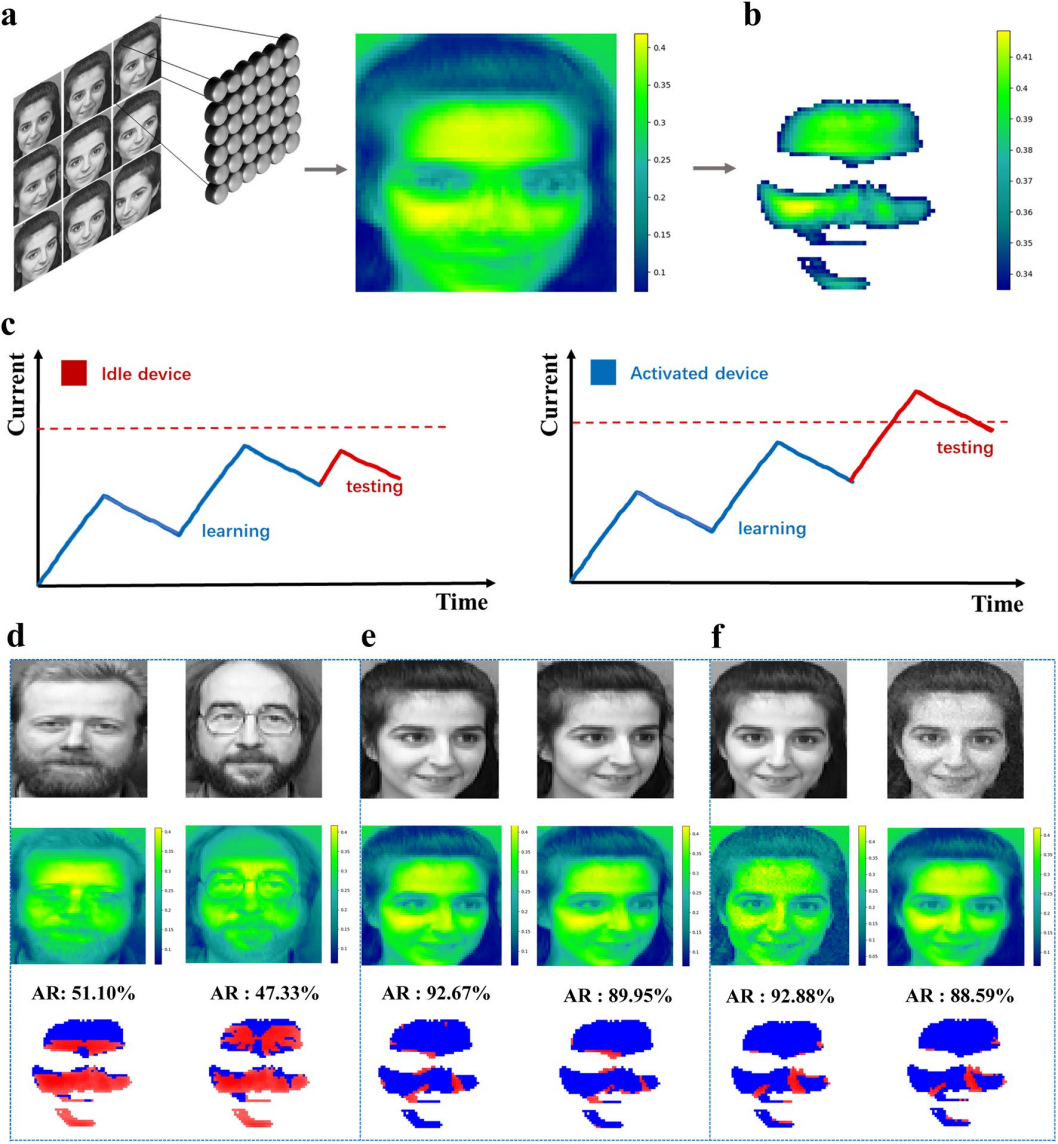
Artificial retina for facial recognition. **a** Illustration of model training of an artificial retina in which optical signals from nine facial images of a woman are fed into a simulated 64 × 64 synaptic array to generate memory current mappings. Locations with higher memory currents correspond to facial regions with higher light reflection. **b** Selection of a subset of synaptic arrays for monitoring facial recognition. **c** Execution conditions in the model that delineate active and idle states. **d** Demonstration of male facial recognition showed a low activation rate of male facial recognition. The AR in the figure represents the activation rate. **e** and **f** Demonstrations of female facial recognition. The results show that the activation rate of facial recognition for some target females is relatively high, with the left side representing noise-free recognition and the right side representing noisy recognition

Based on the experimental results of human visual image memory function, the photosynaptic device is further utilized to simulate the recognition process of human face by human visual system. By normalizing the optical signals (grayscale intensity) of facial features in the images to fit the detection range of the synaptic device, the synaptic array was first trained on nine female grayscale facial images showing different facial expressions and different angles, and the memory current mapping was successfully extracted from them (Figs. 5a and S12). A selected subset of the arrays was used as a facial recognition model due to its high memory current (Fig. 5b), which utilizes the transient light response current of the device at different irradiation times (Fig. S13) to predict facial features. In this model, each synaptic device can adaptively adjust its response according to the execution conditions. As shown in Fig. 5c, when a synapse receives a repetitive visual signal of a female facial image, its transient photoresponse current will exceed the preset execution threshold and be activated, if it is not the target image, it will remain idle. In addition, we tested the vision system for face recognition using six example face images. The results show that the activation rate for male facial images is less than 70% (Fig. 5d), while the activation rate for specific female facial images is more than 92%, showing high recognition accuracy even under noise interference (Fig. 5e, f). This shows that the artificial retina model not only accurately recognizes faces, but also has good noise immunity, opening new application possibilities for the development of AI visual perception systems [37].

**Fig. 6:**
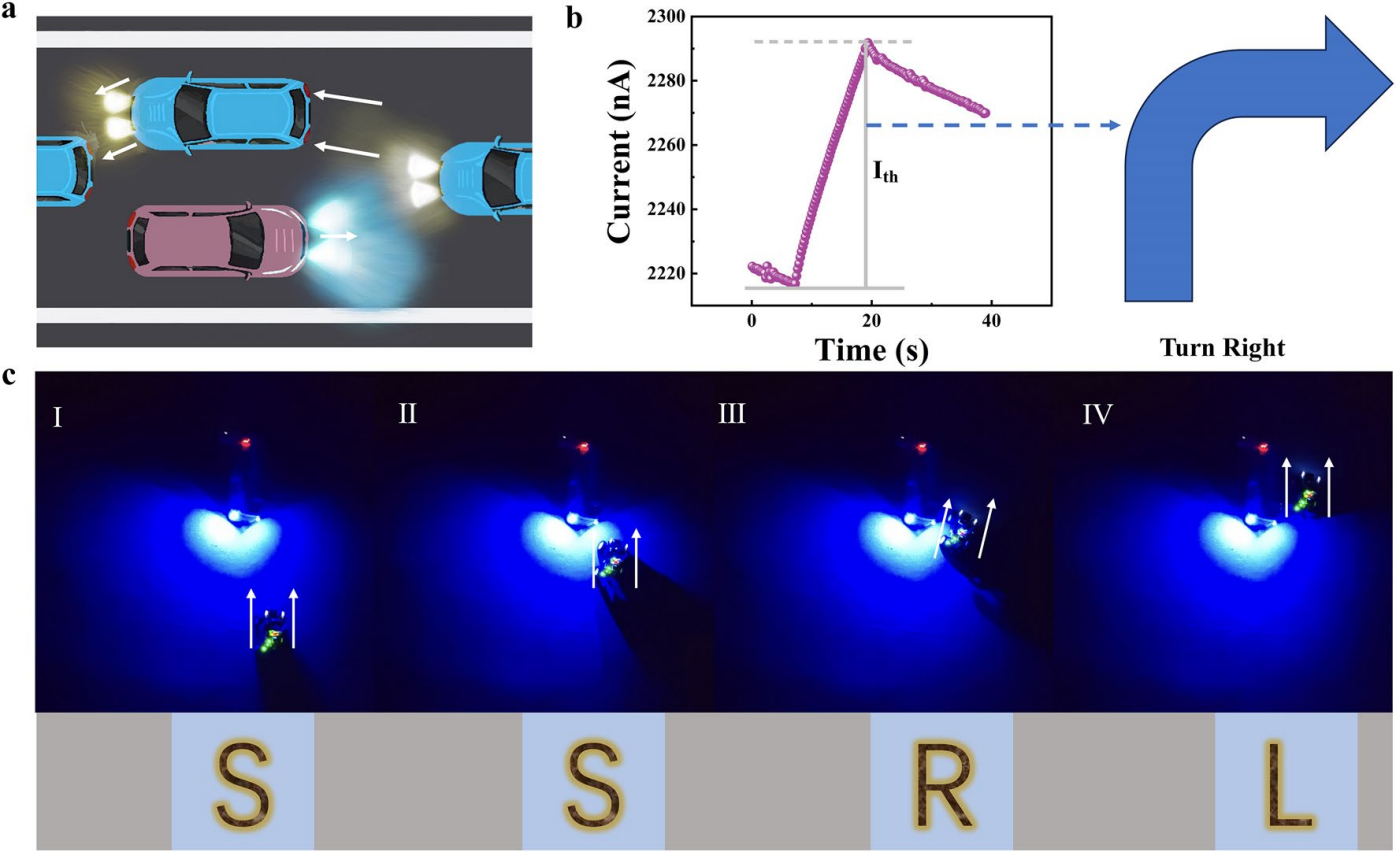
Realization of bidirectional rendezvous system. **a** Bidirectional rendezvous schematic. **b** Linear synaptic weights (and synaptic currents) output by the device in real time, and the meeting behavior is fired when the threshold interval is reached. **c**   Nighttime car meeting demonstration, which mainly shows the “Straight (S),” “S,” “Right (R),” “Left (L)” four states

To further verify the visual perception ability of optical synapses, we designed an unmanned driving system and realized the smart rendezvous function by photosynaptic linear increasing response characteristics, which proved the potential of photosynaptic devices for the application in smart driving system (Fig. S14). Under continuous illumination conditions, the device shows good linear optical response characteristics and is not easy to reach saturation, which is crucial for the accuracy of signal detection in unmanned systems. The driverless car rendezvous control system, the system is in the “Straight” (S) state when two cars are traveling in opposite directions at night, and then gradually approaching each other, the synaptic weights of the optical synapses are gradually increased by sensing the enhanced light intensity (Fig. 6a). When the synaptic current reaches the preset threshold (Fig. 6b), it is processed by the amplifier and the microcontroller, the system sends a “right turn” command to the vehicle via Bluetooth, which guides the vehicle to enter the “Right” (R) state to perform the meeting action. After that, the vehicle drives out of the light range, the current decays below the threshold value, and the vehicle performs the action of “turn left,” i.e., the “Left” (L) state, and finally completes the meeting safely. The whole process is shown in Fig. 6c, and the video of the rendezvous can be seen in the reference information (Video S1). At the end of the whole process, the light disappears and the synaptic weights of the optical synapses devices gradually return to below the set threshold, ensuring the reliability and reusability of the system. This intelligent control not only improves driving safety, but also demonstrates the application prospect of optical synapses devices in modern transportation.

## Conclusions

In this work, we report an artificial optoelectronic memristor device based on TiN/CeO_2_/ZnO/ITO/Mica. Our device exhibits extremely linear (0.996) conductance modulation and low energy consumption (~ 187 pJ) and successfully simulates synaptic properties such as PPF, PPD, STM, LTM, “learning from experience” behavior of a single device, and “Pavlovian” behavior. We have achieved stable optical sensing performance under different lighting conditions. In particular, the image memory function of human vision was simulated by utilizing an ultra-long forgetting time, and the memory time was as long as 8000 s after repeating the observation of an image for 10 times, showing its excellent memory retention ability. Based on the linear conductance modulation capability of the device under different irradiation durations, we also designed and simulated the facial recognition function, which achieved a high facial feature extraction activation rate of 92.88% without relying on complex artificial neural networks, and demonstrated a certain degree of noise immunity. More importantly, with the help of the linear growth characteristics of its optical response, a hardware system for nighttime rendezvous behavior of unmanned vehicles was designed to realize the rendezvous behavior accomplished by using the synaptic behavior of the photoelectric memristor, which ensures the accuracy and reliability of the rendezvous process, which provides a more powerful support for the application in the aspect of the unmanned intelligent system. This work shows that TiN/CeO_2_/ZnO/ITO/mica-based artificial photoelectrical synaptic devices can successfully mimic biological visual memory systems and effectively broaden the scope of applications of artificial intelligence.

## Supplementary Information

Below is the link to the electronic supplementary material.

Supplementary file1 (DOCX 3415 KB)

Supplementary file2 (MP4 1815 KB)
