# Supplementary material for: Low Energy Consumption Photoelectric Memristors with Multi-Level Linear Conductance Modulation in Artificial Visual Systems Application
Source: Nanomicro Lett. 2025 Jul 1;17:317. doi: 10.1007/s40820-025-01816-y (PMC12214075; doi:10.1007/s40820-025-01816-y)
Supplement: Supplementary file 1 — Supplementary file1 (DOCX 3415 KB) [file 40820_2025_1816_MOESM1_ESM.docx]

Supporting Information for

**Low Energy Consumption Photoelectric Memristors with Multi-Level Linear Conductance Modulation in Artificial Visual Systems Application**

Zhenyu Zhou ^1^, Zixuan Zhang ^1^, Pengfei Li ^1^, Zhiyuan Guan ^1^, Yuchen Li ^1^, Xiaoxu Li^1^, Shan Xu^1^, Jianhui Zhao ^1^, and Xiaobing Yan ^1^*

^1^ College of Electron and Information Engineering, School of Life Sciences, Institute of Life Science and Green Development, Key Laboratory of Brain-Like Neuromorphic Devices and Systems of Hebei Province, Hebei University, Baoding 071002, People's Republic of China

*Corresponding author. E-mail: [yanxiaobing@ime.ac.cn](mailto:yanxiaobing@ime.ac.cn) (Xiaobing Yan)

**Supplementary Figures**


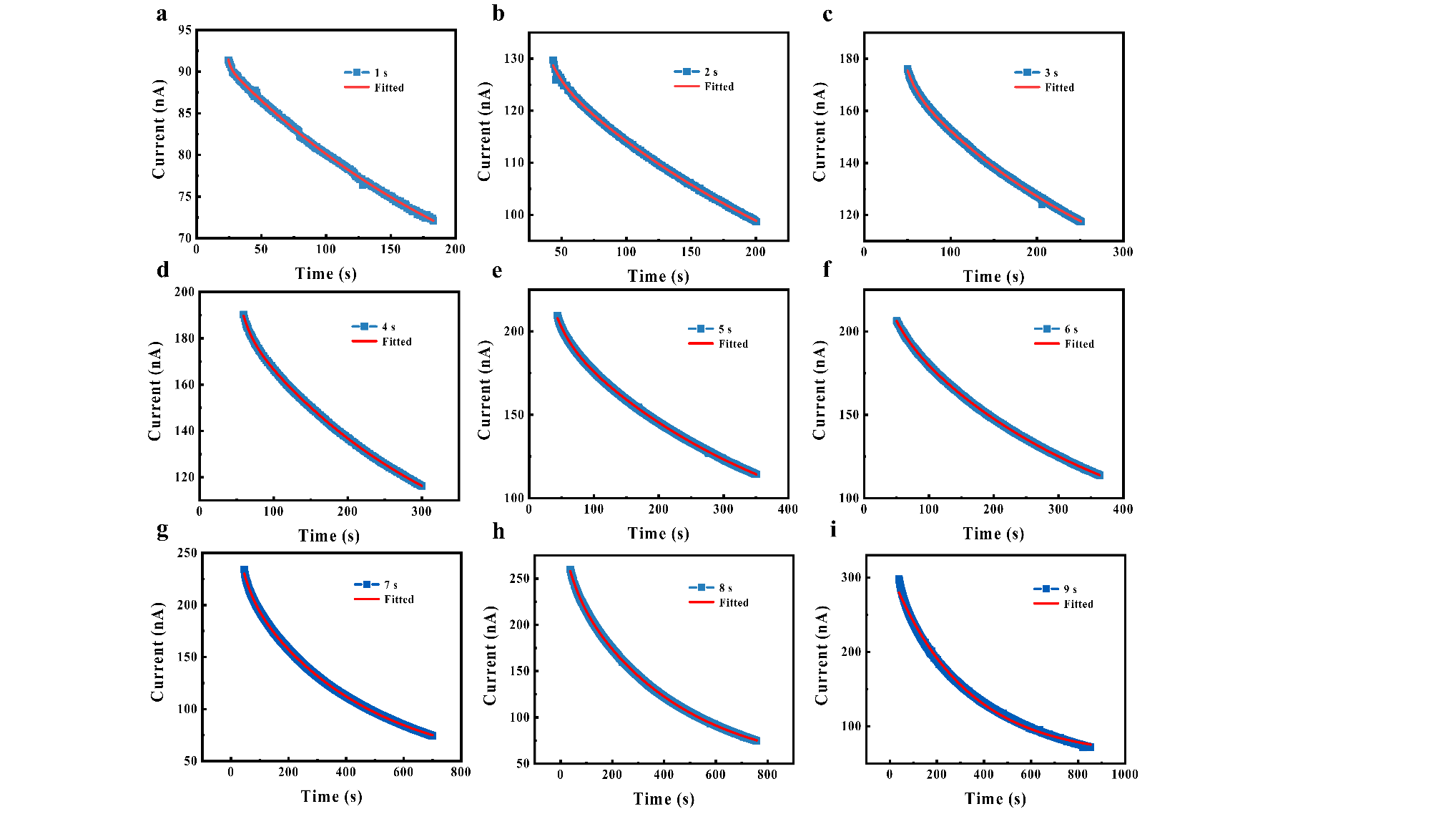


**Fig. S1** **a-i** Photocurrent attenuation response (blue curve) and fitting curve (red line) of the device under different illumination durations





**Fig. S2** Stability of 9 random device-to-device under different light pulses


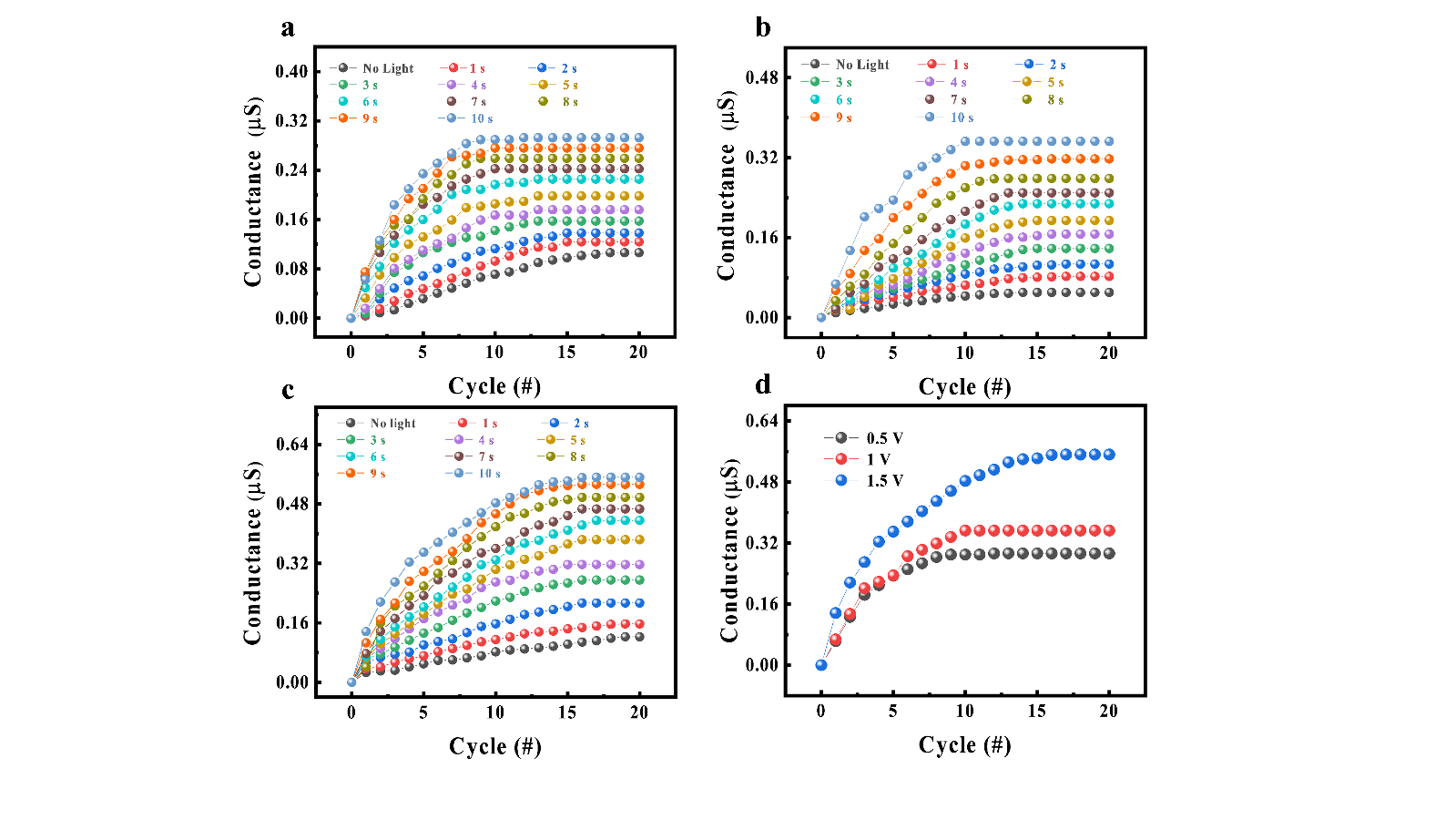


**Fig. S3** The effect of changing the amplitude of the pulse and the illumination time on the conductivity of the device. **a-c** The effects of different light exposure times on device conductivity under different pulse amplitudes (0.5 V, 1 V, and 1.5 V); **d** The effect of different amplitudes on device conductivity after irradiation for the same time (10 s)


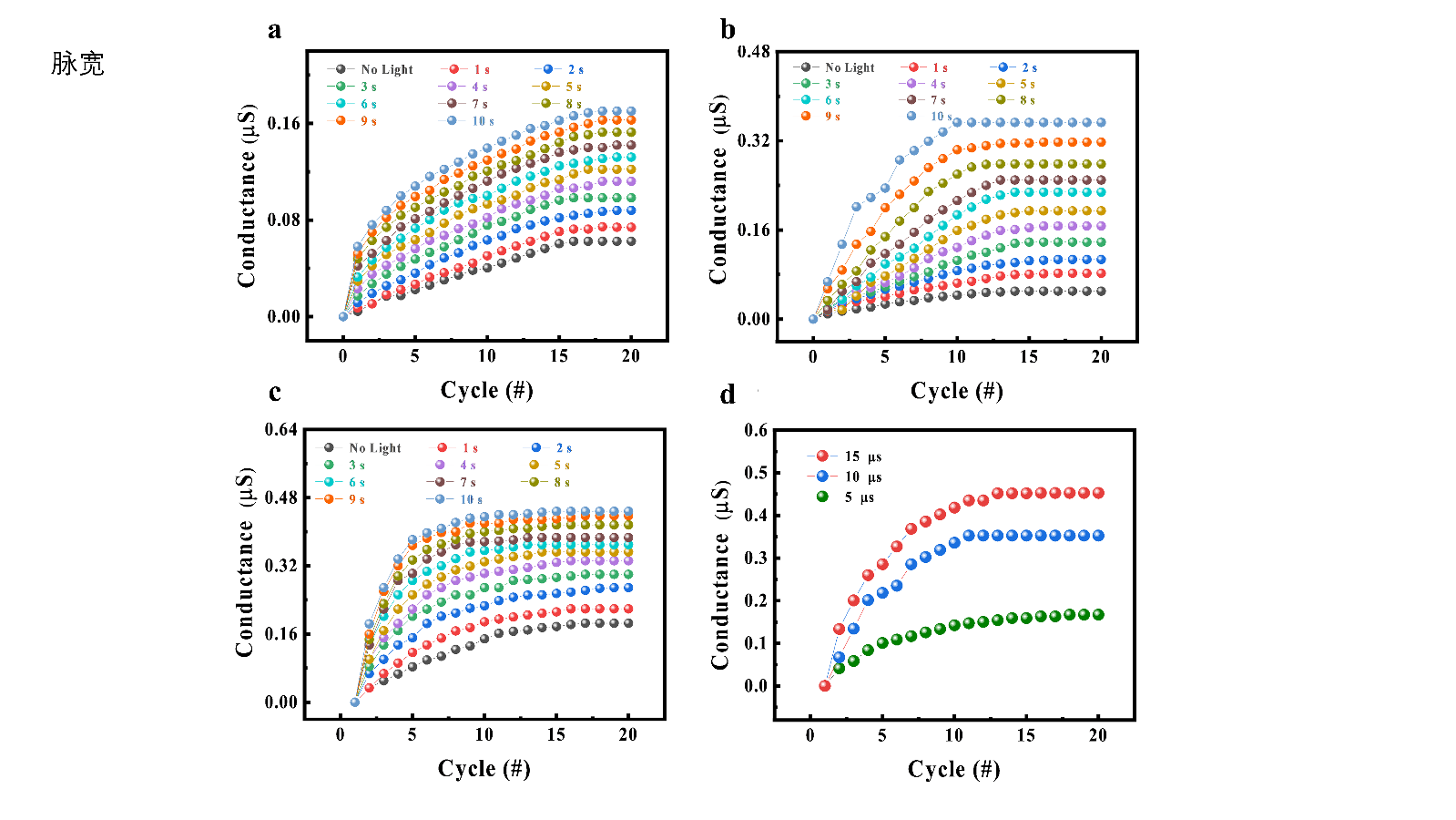


**Fig. S4** The effect of changing the pulse width and illumination time on conductivity. **a-c** The effects of different light exposure times on conductivity under different pulse widths (5 μs, 10 μs, and 15 μs); **d** The effect of different pulse widths on conductivity after irradiation for the same time (10 s)


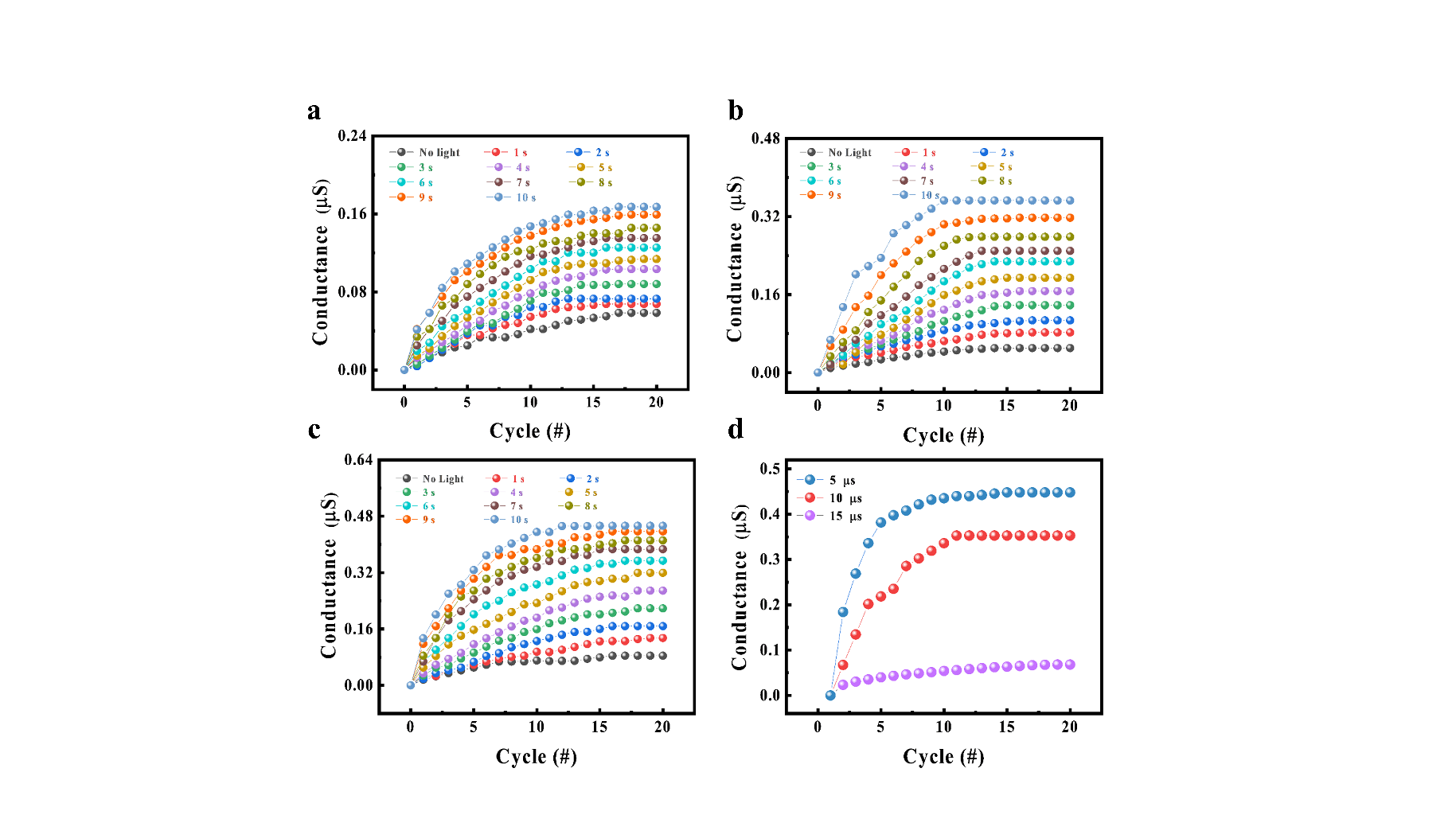


**Fig. S5** The effect of changing the pulse interval and illumination time on conductivity. **a-c** The effects of different light exposure times on conductivity at different pulse intervals (5 μ s, 10 μ s, and 15 μ s); **d** The effect of different pulse intervals on conductivity after irradiation for the same time (10 s)


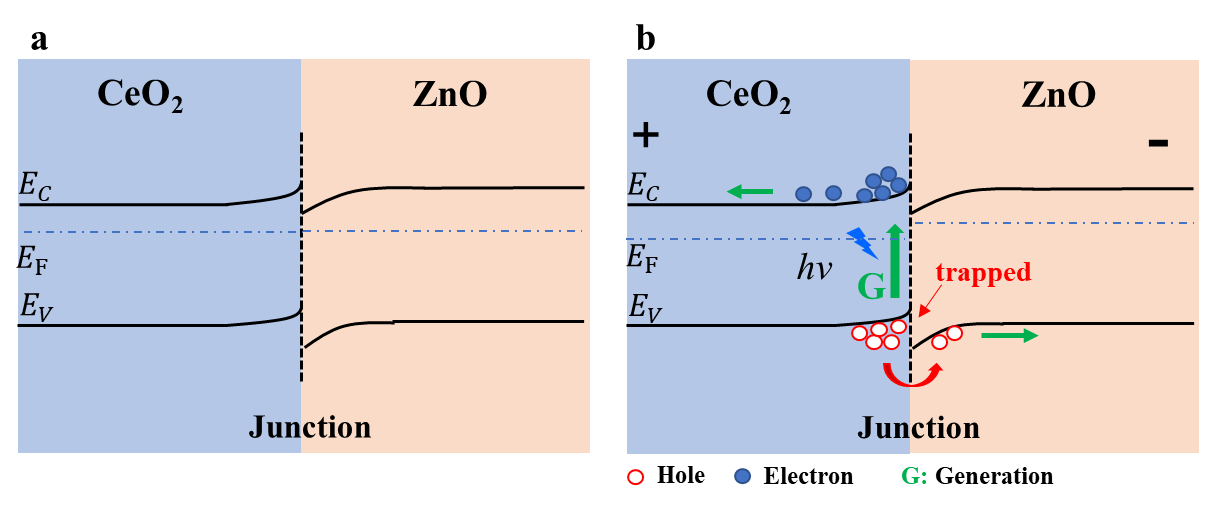


**Fig. S6** **a** Band diagram in the state of thermal equilibrium; **b** Band diagram under photoelectric action


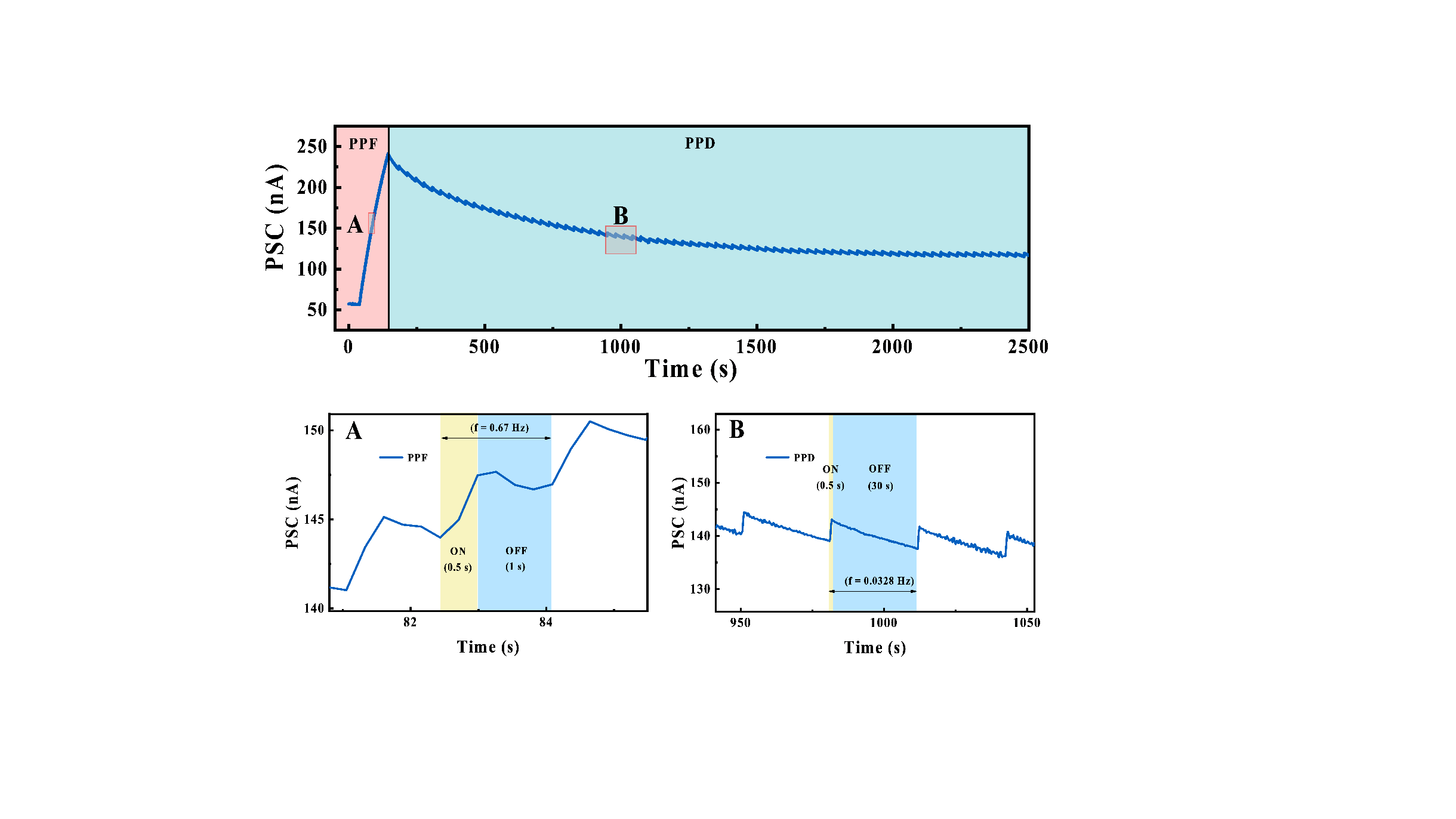


**Fig. S7** Extraction magnification of frame A and frame B of PPF and PPD in manuscript. PPF obtained at a pulse width of 0.5 s and a time interval of 1 s; b PPD obtained at a pulse width of 0.5 s and an increasing time interval of 30 s


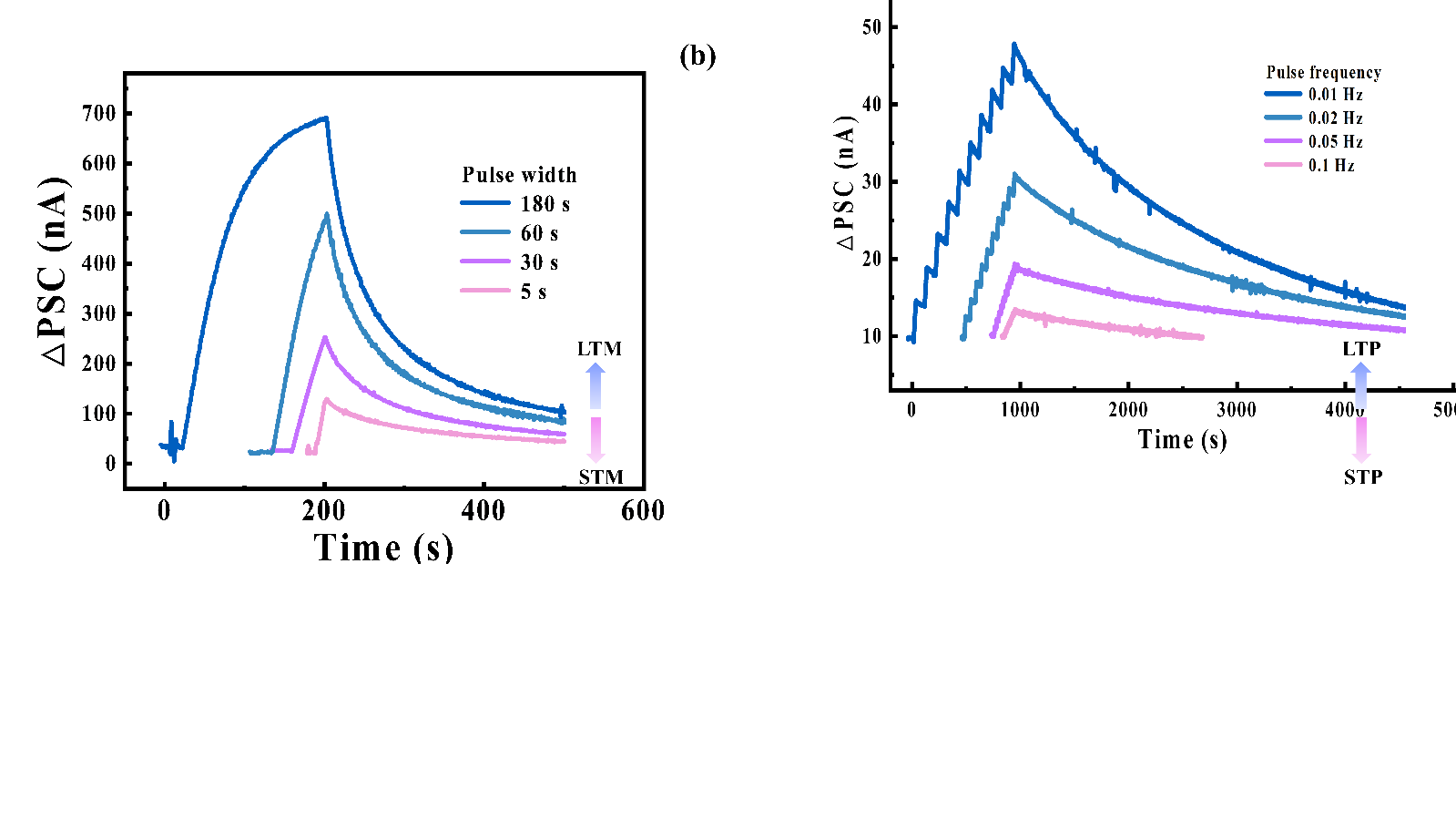


**Fig. S8** Complete the transition from STM to LTM by modulating the pulse width time of light pulses (5 s to 180 s)





**Fig. S9** The transition from STM to LTM is achieved by modulating the number of light pulses (10-50), with the illustration showing the response form of the device when increasing the light pulse irradiation


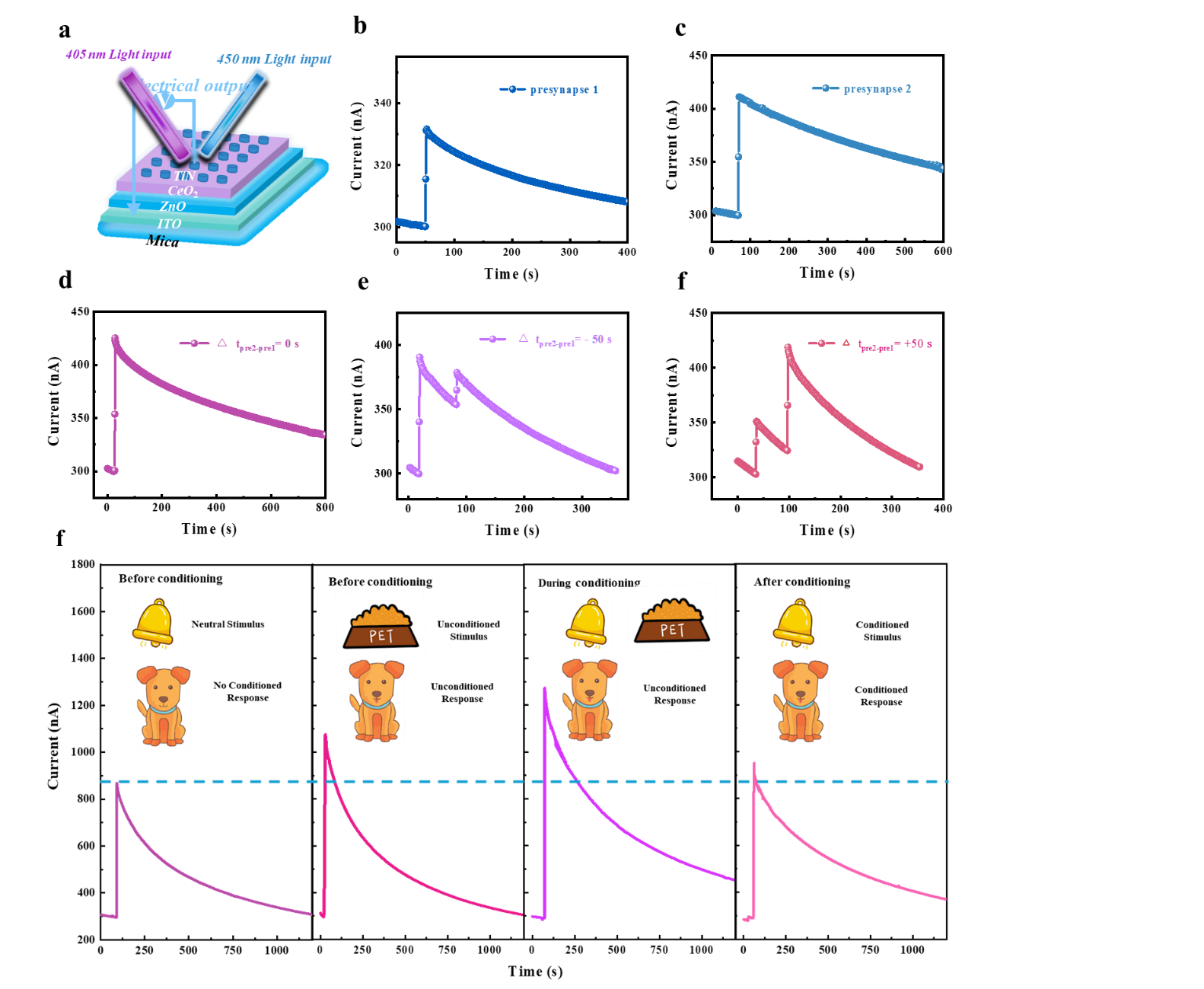


**Fig. S10** **a** Schematic diagram of applying laser irradiation with wavelengths of 450 nm and 405 nm to the device; **b** PSC1 of the device after 450 nm wavelength laser irradiation; **c** PSC2 of the device after 405 nm wavelength laser irradiation; **d-f** PSC response of devices under different illumination intervals, with intervals of 0 s, −50 s, and +50 s, respectively. **g** Pavlov's Classic Reflection Experiment. Using 450 and 405 nm as neutral and unconditioned stimuli, the blue dashed line represents the threshold, set to 900 nA. After training, the conditioned stimulus produces an effective response, with a PSC of 956 nA.

Changing the number of pulses and the time between two consecutive pulses can greatly affect the response of the device, as shown in Fig. S10a. When the device is irradiated with a light source with wavelengths of 405 nm and 450 nm respectively, the response is referred to as "presynaptic 1" when using a 450 nm light source with a pulse width of 1 second and a power of 50 mW. The changes in PSC1 can be observed as shown in Fig. S 10b. Under the same conditions, using a 405 nm light source to irradiate and excite "presynaptic 2", its PSC2 is 70% larger than PSC1, as shown in Fig. S10c. When both presynaptic 1 and presynaptic 2 are triggered simultaneously, the amplitude of PSC undergoes the maximum change, as shown in Fig. S10d. This is because the photogenerated carriers of the second optical pulse are added to the photogenerated carriers of the first optical pulse. However, this response can be adjusted by changing the time interval Δ t between peaks, as shown in Fig. S S9e and 9f, where different time intervals of light pulse triggering devices adjust different PSC responses.

By irradiating with a light pulse with a wavelength of 450 nm (pulse width of 10 s and intensity of 50 mW) to initiate neutral stimulation (Fig. S10g), the PSC reached 892 nA, which can be considered as the initial stage of ringing. Using a light pulse with a wavelength of 405 nm (pulse width of 10 s and intensity of 50 mW) as an unconditional stimulus for feeding, the maximum PSC of 1195 nA was obtained by jointly irradiating with wavelengths of 450 nm and 405 nm to mimic feeding and ringing situations. It can be observed that the PSC generated solely by neutral stimuli of ringing is smaller than the response to unconditioned stimuli, which is set within a threshold range of 900 nA. After the conditioning stage, the neutralizing stimulation of the bell resulted in a PSC of 956 nA, exceeding the threshold of 900 nA. Under 450 nA illumination, PSC above the threshold was obtained due to an increase in the number of intrinsic charge carriers after a series of adjustments were made to the device. It can be seen that after adjusting the device, the Pavlov experiment was successfully simulated.


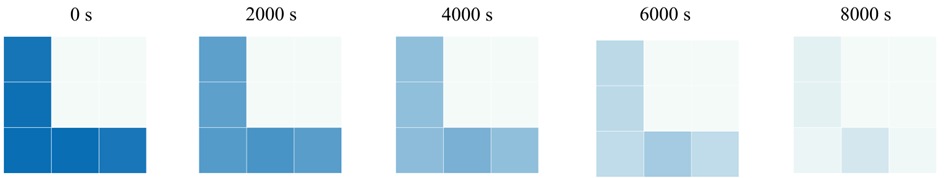


**Fig. S11** Observing the optical response image mapping forgetting time after 10 times


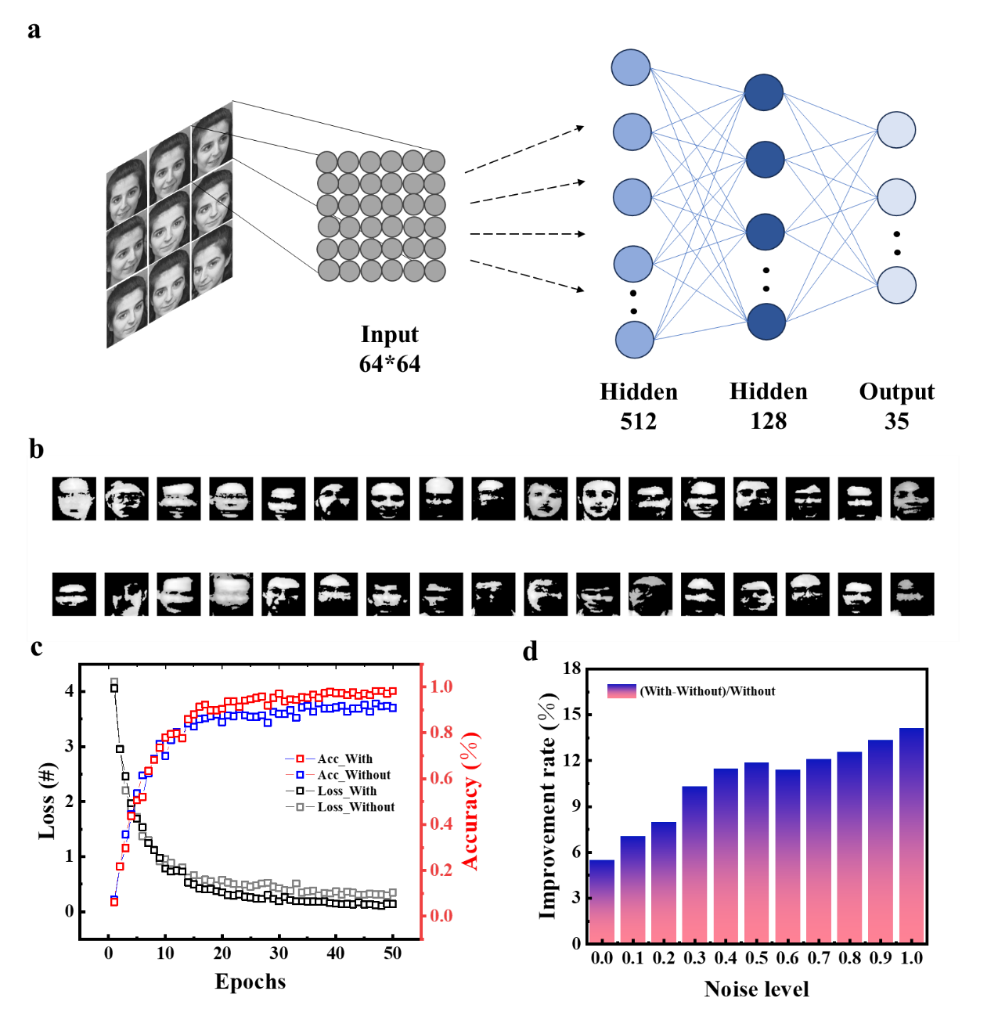


**Fig. S12** Model training of the artificial retina. **a**, the architecture schematic diagram of the input image (64*64) to the multi-layer perceptron (MLP), including the input layer, two hidden layers (512 and 128 neurons respectively), and the output layer (34). **b**, facial feature images after adaptive preprocessing. **c**, the Loss and Accuracy curves with and without memristor adaptive preprocessing during the training process, reflect the optimization effect of preprocessing on the model performance. **d**, under different noise levels, the influence of memristor adaptive preprocessing on the model improvement rate demonstrates extremely excellent anti-noise ability

Fig. S12 shows the model training and performance analysis based on the adaptive processing of memristors. This model is based on the ORL face dataset (34 classes, with 10 grayscale images (64×64) in each class), and is input after normalization, memristor conversion and adaptive threshold processing. The model is a multi-layer perceptron (MLP). The input layer is flattened to 4096 dimensions, containing two hidden layers (512 and 128 neurons, using ReLU, BatchNormalization and different proportions of Dropout), and the output layer has 34 neurons (activated by Softmax). Hyperparameters: Adam optimizer (learning rate (1e-3)), batch size 16, 50 rounds of training, 10% of the training set is taken as the validation set, the loss function is Categorical crossentropy, and the accuracy rate is evaluated. Fig. S12a signal input to the architecture of MLP, Fig. S12b after adaptive preprocessing for 34 kinds of facial features of the image. Fig. S12c shows that the adaptive preprocessing with memristors (93.58%) has a higher accuracy rate than no preprocessing (88.90%), and the loss reduction is better, indicating that preprocessing effectively optimizes the model performance. Fig. S12d indicates that with the increase of noise level, the model improvement rate of memristor adaptive preprocessing rises, highlighting its strong anti-noise ability. Photoelectric memristor devices not only achieve photoelectric conversion, but also extract image features based on threshold algorithms, reduce the amount of inference data for network training, improve convergence, facilitate network training, effectively enhance the model's processing ability for complex datasets, and provide strong support for dealing with variable conditions in practical applications.

**
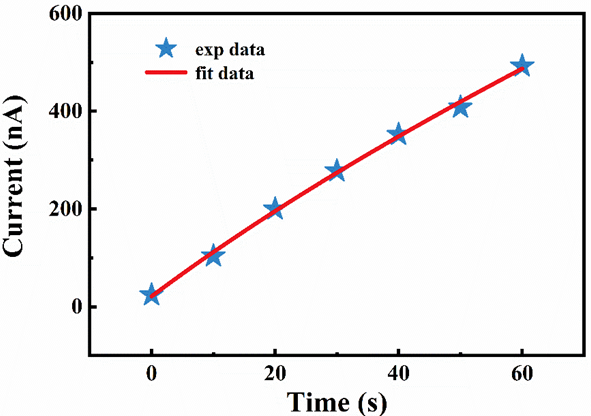
**

**Fig. S13** Photoinduced non-volatile current as a function of illumination time
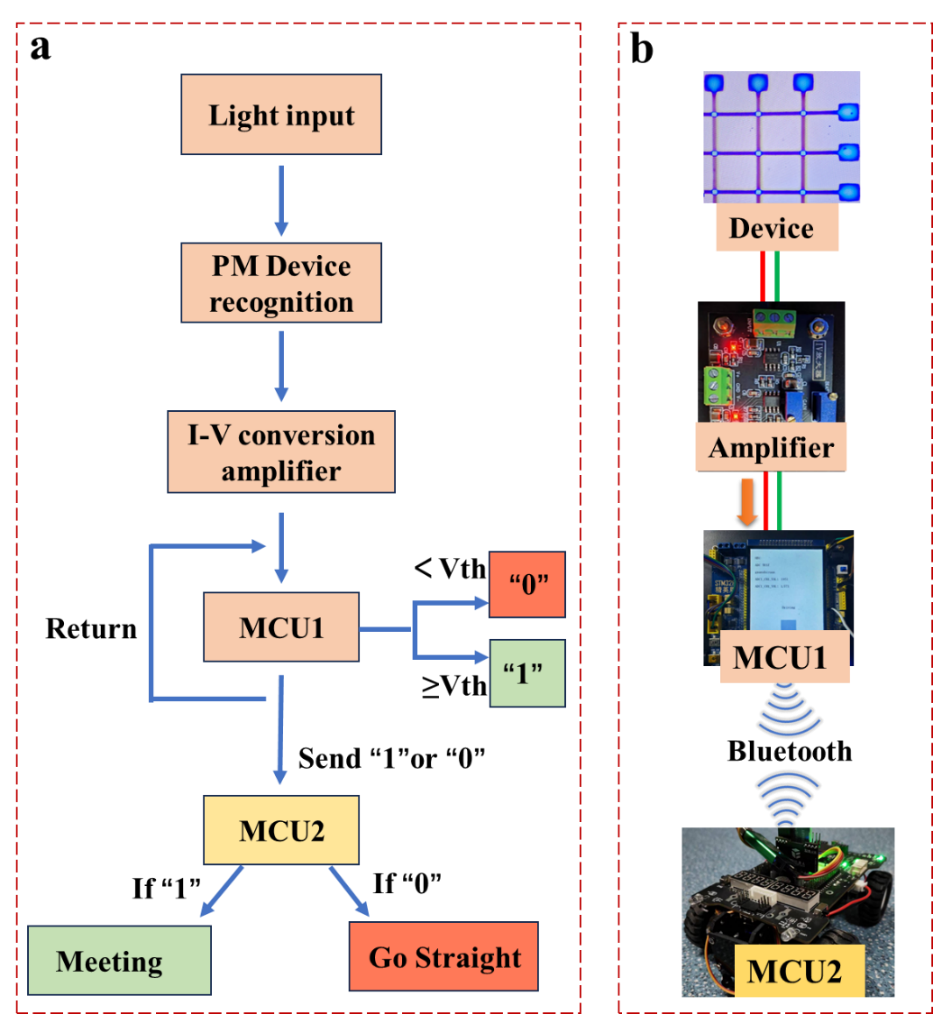


**Fig. S14** The judgment flowchart of the oncoming vehicle application and the optical images of each part of the system

**Table S1** Comparison of synaptic characteristics of neuromorphic devices.

| Materials | Light wavelength | E (J) | EPSC | PPF | STM | LTM |
| --- | --- | --- | --- | --- | --- | --- |
| a-GaO*_x_* [1] | 365nm | ~5×10^-10^ | No | Yes | Yes | No |
| GaN/Ga_2_O_3_/GaN [2] | 254nm,365nm | ~5.8×10^-10^ | No | Yes | No | No |
| ITO-chitosan [3] | 400nm | ~4.2×10^-6^ | Yes | Yes | No | No |
| Black phosphor [4] | 280-660nm | ~9.24×10^-10^ | Yes | Yes | Yes | Yes |
| Graphene/diamond [5] | 470nm | ~4.4×10^-10^ | Yes | Yes | Yes | Yes |
| BP–GOQD [6] | 365nm | ~1.7×10^-9^ | Yes | Yes | Yes | Yes |
| (PEA)_2_SnI_4_ [7] | 470nm | ~3.6×10^-8^ | Yes | Yes | Yes | Yes |
| **This work** | 405nm,450nm | ~1.87×10^-10^ | Yes | Yes | Yes | Yes |

**Table S2** The decay time constants of photocurrent under different irradiation times τ 1 and τ 2

| t (s) | 1 s | 2 s | 3 s | 4 s | 5 s | 6 s | 7 s | 8 s | 9 s |
| --- | --- | --- | --- | --- | --- | --- | --- | --- | --- |
| τ1 (s) | 2.2 | 10.2 | 11.5 | 12.8 | 23.0 | 28.1 | 37.1 | 47.0 | 54.5 |
| τ2 (s) | 227.0 | 260.7 | 262.5 | 277.1 | 352.0 | 331.4 | 414.8 | 417.3 | 418.6 |

The memory time of the human brain is closely related to the learning duration, and this phenomenon is also reflected in the current response of TiN/CeO_2_/ZnO/ITO/Mica memristors. Fit the photocurrent attenuation curve of the pulse response at different irradiation times in Fig. S1 using a double exponential function. The fitting function is:

$$I_{\text{d}}\text{= }y_{0}\text{+}A_{1}\text{exp}\left(-\text{Δ}t\text{/τ}_{1}\right)\text{+}A_{2}\text{exp}\left(-\text{Δ}t\text{/τ}_{2}\right)$$

The decay time constants τ _1_ and τ _2_ generated from this are shown in Table S2. Within the duration of illumination from 1 to 9 s, τ _1_ increases from 2.2 to 54.5 s, and τ _2_ increases from 227.0 to 418.6 s, where τ _1_ and τ _2_ correspond to shallow and deep level captured states, respectively.

**Supplementary References**

1. Z. Zhang, X. Zhao, X. Zhang, X. Hou, X. Ma et al., In-sensor reservoir computing system for latent fingerprint recognition with deep ultraviolet photo-synapses and memristor array. Nat. Commun. **13**(1), 6590 (2022). <https://doi.org/10.1038/s41467-022-34230-8>
2. S. Feng, J. Li, L. Feng, Z. Liu, J. Wang et al., Dual-mode conversion of photodetector and neuromorphic vision sensor *via* bias voltage regulation on a single device. Adv. Mater. **35**(49), 2308090 (2023). <https://doi.org/10.1002/adma.202308090>
3. Y.B. Guo, L.Q. Zhu, W.T. Gao, Z.Y. Ren, H. Xiao et al., Low-voltage protonic/photonic synergic coupled oxide phototransistor. Org. Electron. **71**, 31–35 (2019). <https://doi.org/10.1016/j.orgel.2019.04.038>
4. T. Ahmed, S. Kuriakose, E.L.H. Mayes, R. Ramanathan, V. Bansal et al., Optically stimulated artificial synapse based on layered black phosphorus. Small **15**(22), 1900966 (2019). <https://doi.org/10.1002/smll.201900966>
5. Y. Mizuno, Y. Ito, K. Ueda, Optoelectronic synapses using vertically aligned graphene/diamond heterojunctions. Carbon **182**, 669–676 (2021). <https://doi.org/10.1016/j.carbon.2021.06.060>
6. J. Zeng, L. Ding, S. Yuan, Z. Feng, L. Dong et al., A passivation strategy assisting a robust and low consumption power BP-based optical synaptic device for neural computing. J. Mater. Chem. C **12**(15), 5529–5537 (2024). <https://doi.org/10.1039/D3TC04645A>
7. Y. Sun, L. Qian, D. Xie, Y. Lin, M. Sun et al., Photoelectric synaptic plasticity realized by 2D perovskite. Adv. Funct. Mater. **29**(28), 1902538 (2019). <https://doi.org/10.1002/adfm.201902538>
